# Braithwaite’s Retrospect

**Published:** 1861-08

**Authors:** 


Braithwaite's *Retrospect*, Part XLIII, with 352 solid pages, fully maintains its well-earned reputation; and the physician, unable to subscribe for the leading foreign periodicals, can make no more economical investment, than by subscribing for the *Retrospect*,—that is, if he would put himself *en rapport* with the improvements and progress of his profession, in its every branch. The diseases of women, having given rise, of late, to, perhaps, more attention than has been devoted to any other special subject, the editor, peculiarly fitted for the task, by his position as lecturer on *Obstetric Medicine* in the Leeds School, makes a special report and commentary on the valuable and interesting papers thus drawn forth during the past six months; and this, it is proposed to continue from time to time. This number also contains the index for the volume, and the usual useful synopsis of the most practical articles, showing, briefly and comprehensively, the most important indications of treatment, published by different writers within the last half year.

W. A. Townsend, 39 Walker street, New York, is the American publisher; and W. B. Keen, 148 Lake street, Chicago, the Illinois agent.

